# Effect of Dietary Flaxseed Oil Supplementation on the Redox Status, Haematological and Biochemical Parameters of Horses’ Blood

**DOI:** 10.3390/ani10122244

**Published:** 2020-11-30

**Authors:** Iwona Sembratowicz, Grzegorz Zięba, Ewelina Cholewinska, Anna Czech

**Affiliations:** 1 Department of Biochemistry and Toxicology, Faculty of Animal Sciences and Bioeconomy, University of Life Sciences in Lublin, Akademicka 13, 20-950 Lublin, Poland; iwona.sembratowicz@up.lublin.pl (I.S.); ewelina.cholewinska@up.lublin.pl (E.C.); 2 Department of Biological Basis of Animal Production, Faculty of Animal Sciences and Bioeconomy, University of Life Sciences in Lublin, Akademicka 13, 20-950 Lublin, Poland; grzegorz.zieba@up.lublin.pl

**Keywords:** horses, blood parameters, flaxseed oil, antioxidant, lipids parameters, haematological parameters

## Abstract

**Simple Summary:**

Flaxseed oils have long been used in animal feeding and human nutrition due to the beneficial effects of their biologically active substances, especially omega-3 polyunsaturated fatty acids (PUFA). This study aimed to evaluate the effect of the replacement of soybean oil (SO) with flaxseed oil (FO) in the horses’ diet on biochemical, haematological, and redox status parameters of the blood. We found FO contributed to an improvement of lipid profile, haematological parameters of the blood and enhanced antioxidant defence mechanisms of horses. This suggests that substitution of SO with FO in the diet of horses is beneficial for their health status.

**Abstract:**

This study compared the effect of two dietary vegetable oils on plasma biochemical indices, haematological parameters, and redox status of horses. Forty riding horses (20 mares and 20 stallions) of the Malopolski breed were divided equally into two groups that were similar in terms of age, sex, and body weight (on average 530 ± 30 kg). The horses received soybean oil (SO) or flaxseed oil (FO) in the amount of 25 mL per 100 kg BW/day. After 60 days, blood was collected for biochemical and haematological analyses. The results show that horses receiving FO as compared to the SO group had significantly lower plasma levels of glucose, low density lipoprotein cholesterol, total cholesterol/high-density lipoprotein (HDL) ratio and triacylglycerols, as well as the activities of alanine aminotransferase and alkaline phosphatase. In turn, %HDL-TC and lactate dehydrogenase activity were significantly higher in the FO group. The inclusion of FO in the diet contributed to an increase in antioxidant indices: creatinine, vitamin C, copper, and zinc contents and also superoxide dismutase and catalase activities. The level of the end product of lipid peroxidation, i.e., malonyl dialdehyde, in the FO group as compared to the SO group was significantly lower. Moreover, FO caused an elevation in red blood cell indicators, lymphocyte count and lysozymes. In conclusion, FO exerts a beneficial effect by stimulating antioxidant defence mechanisms of horses and reducing the severity of oxidative stress. FO also improved the lipid profile and haematological parameters of the blood. The replacement of SO by FO is recommended based on these findings.

## 1. Introduction

In horse nutrition, particular focus is placed on the quality of the fat used. In place of popular vegetable oils, such as soybean, rapeseed or maize oil, other valuable oils are used as well, e.g., fish or flaxseed oil. The advantage of flaxseed oil over other vegetable oils stems from its high content of ω-3 polyunsaturated fatty acids (PUFA), especially alpha-linolenic acid (ALA), of which it is the richest plant source [1]. Moreover, flaxseed oil has a highly favourable ratio of ω-6 to ω-3 acids, not exceeding 2:1, while the ratio in soybean oil, for example, is about 10:1 [2]. In the body, ω-6 and ω-3 fatty acids compete as substrates of the same enzyme systems [3]. An excess of ω-6 fatty acids in the diet has been shown to inhibit metabolism of ω-3 fatty acids, which can disturb the homeostasis of the biologically active compounds synthesized from them, which are mutual antagonists [4]. Enrichment of the diet with ω-3 acids, e.g., using flaxseeds or flaxseed oil, may help to suppress inflammatory and allergic reactions, improve the blood lipid profile, and increase tissue sensitivity to insulin [5]. In the diet of horses, flaxseeds or flaxseed oil has been used as a laxative [6] and to treat inflammatory and allergic skin conditions [7]. Apart from its high content of ALA, flaxseed oil contains other health-promoting compounds, such as phytosterols (with an anti-sclerotic effect), carotenoids and phenolic acids (with antioxidant activity) [8]. Lignans present in flaxseeds also exhibit antioxidant properties and additionally function as phytoestrogens [1]. Despite the unquestionable advantages of the use of flaxseed oil in the diet of humans and animals, the introduction of large amounts of PUFA to the diet, especially ω-3 acids, which are particularly susceptible to oxidation, may risk increasing oxidation reactions in the body. This can result in antioxidant imbalance and the onset of oxidative stress [9]. Such a scenario is indicated by research by [3] on horses and by [10] on turkeys. It should be noted, however, that numerous experiments have confirmed the beneficial effect of flaxseed oil on the alleviation of oxidative stress accompanying various disease entities or intoxication [1,11]. A study by Melo et al. [12] on gaited horses revealed that the inclusion of oils rich in ω-3 and ω-6 fatty acids increased their antioxidant capacity and in consequence improved the performance of the animals.

In view of the above, we postulated that the inclusion of flaxseed oil in the diet of horses may enhance antioxidant defence mechanisms and positively influence blood biomarkers; thus, improving their health status. Therefore, the aim of this study was to assess the effect of the replacement of soybean oil with flaxseed oil in the diet of horses on redox status and haematological and biochemical parameters of the blood.

## 2. Material and Methods

### 2.1. Animals and Experimental Design

The study was conducted in March and April (for 60 days) on a farm in South-eastern Poland. Forty riding horses of the Malopolski breed were selected for the study.

The study group consisted of 20 mares and 20 stallions. The horses were aged 6 to 10 years. They were divided into two groups that were similar in terms of age, sex, and body weight (on average 530 ± 30 kg), with 20 horses in each. All horses were clinically healthy and were kept in identical, very good welfare conditions. They were housed in individual 3 × 3 m stalls. During the experiment, the horses performed light work consisting of daily lunging (for about 20–30 min).

Every day the horses received a basal feed ration consisting of meadow hay (1.3 kg/100 kg BW/day) and crushed oats (0.54 kg/100 kg BW/day) in two equal portions (at 8 am and 5 pm). In addition, they received a mineral and vitamin supplement in the amount of 20 g/100 kg BW during morning feeding and wheat straw as litter (8 kg/day/stall). The mineral and vitamin supplement was added to the oats during the morning feeding. One kilogram of the supplement contained 160 g calcium; 40 g phosphorus; 20 g sodium; 40 g magnesium; 500,000 IU vitamin A; 50,000 IU vitamin D_3_; 10,000 mg vitamin E; 5000 mg vitamin C; 400 mg vitamin B_1_; 400 mg vitamin B_2_; 250 mg vitamin B6; 1000 mg vitamin B_12_; 5000 mg niacin; 20,000 mcg biotin; 200 mg folic acid; 200 mg Ca-D pantothenate; 10,000 mg choline chloride; 2000 mg iron; 1000 mg zinc; 1000 mg zinc chelate; 500 mg manganese; 500 mg manganese chelate; 250 mg copper; 250 mg copper chelate; 30 mg iodine; 10 mg selenium; and live yeast cultures (*Saccharomyces cerevisiae* SC 47)—5 × 10^10^ CFU. The animals had continual access to drinking water and spent at least 7 h a day in paddocks.

The composition of the diets satisfied the daily requirements for energy, protein, minerals, and vitamins for horses, based on the average food ration for the species according to National Response Corporation [13]. The basal diet was established for a horse with a body weight of 530 ± 30 kg performing light work. The basal diet did not cover the requirement for energy. Energy was supplied by adding oil as an experimental factor. The content of protein in the feed ratio was 143 g/100 kg BW/day, and the energy level was 17.5 MJ/100 kg BW/day.

The factor differentiating the groups was the kind of oil supplementing the basal diet in the amount of 25 mL/100 kg BW/day. Soybean oil was used in the control group (SO group), while the animals in the experimental group received flaxseed oil (FO group). The fatty acid composition of the oils is given in Table 1. The oil was administered following morning feeding, directly to the muzzle with a syringe by an individual that cared for the horses on a daily basis.

### 2.2. Chemical Analyses of Oils

Prior to the start of the experiment, the oils used were analysed for their fatty acid profile, content of alpha-, gamma- and delta-tocopherol, and DPPH^•^ free radical scavenging activity.

The fatty acid profile of the oils was analysed by gas chromatography according to PN-EN ISO 5508:1996 and PN-EN ISO 5509:2001. The Varian 450-GC gas chromatograph with a flame ionization detector (FID) was used for the analysis.

Tocopherol was assayed by high-performance liquid chromatography (HPLC) according to PN-EN ISO 9936:2016-05. The content of various forms of tocopherol was determined by reversed-phase HPLC according to PN-EN 12822:2002.

DPPH^•^ free radical scavenging activity was measured by the method developed by Bocco et al. [14]. First, a 0.05 mL aliquot of the previously diluted sample was added to 1.95 mL of a 0.06 mmol/L DPPH^•^ solution in methanol and mixed. After 25 min, the decrease in the absorbance was measured by spectrophotometry at 515 nm (Unicam 5625 UV/VIS Spectrophotometer, RIGAL BEN NETT, East Yorkshire, UK). The exact DPPH^•^ concentration remaining in the solution was calculated from a calibration curve.

Analyses of oils were performed in three replications.

### 2.3. Blood Analysis

After 60 days of administration of the experimental factor, about 10 mL of blood was drawn from the external jugular vein into heparinized tubes. Blood was collected before morning feeding at 6:00 am. As the blood was taken by a veterinary surgeon for routine testing, approval of the ethics committee was not required for this procedure. The analyses were performed immediately after the blood was collected.

The following haematological parameters were determined in whole blood: haematocrit (HCT), haemoglobin content (Hb), red blood cell count (RBC), mean corpuscular volume (MCV), mean corpuscular haemoglobin (MCH) and mean corpuscular haemoglobin concentration (MCHC), as well as the white blood cell count (WBC) and the percentage composition of white blood cells (leukogram), i.e., the percentage of neutrophils (NEU), lymphocytes (LYM), and the sum of monocytes, eosinophils and basophils (MID). The determinations were made in an ABACUS-Vet analyser.

Biochemical indices, i.e., glucose, total protein (TP), uric acid (UA), creatinine (CREAT), total cholesterol (TC), high-density lipoprotein (HDL) cholesterol, and triacylglycerols (TG), were determined in the plasma by spectrophotometry using Cormay monotests. The content of the low-density lipoprotein cholesterol fraction (LDL) was calculated.

Activity of alanine aminotransferase (ALT), aspartate aminotransferase (AST), alkaline phosphatase (ALP) and lactate dehydrogenase (LDH), as well as the content of phosphorus, calcium, magnesium, cuprum, zinc, and iron were determined by spectrophotometry. The concentrations of phosphorus (P), calcium (Ca), magnesium (Mg), copper (Cu), zinc (Zn), and iron (Fe) in the blood plasma were determined in the Central Apparatus Laboratory, University of Life Sciences in Lublin, Poland by the flame atomic Absorption Spectrometry technique on a UNICAM 939 spectrometer (Cecil Instruments Ltd., Milton Technical Centre, Cambridge, UK).

Serum lysozyme activity was determined by the turbidimetric method [15]. In addition, the blood plasma was analysed for the level of malondialdehyde (MDA) as an end product of tissue lipid oxidation according to Salih et al. [16]. Spectrophotometric assays were also used to test the blood plasma samples for activity of antioxidant enzymes: superoxide dismutase (SOD) by the adrenaline method, with a modification of the wavelength to 320 nm to increase the selectivity of transient reaction products [17], and catalase (CAT) according to Clairborne [18]. Total antioxidant potential (ferric reducing ability of plasma—FRAP) and plasma concentrations of vitamin C were determined according to Benzie and Strain [19].

### 2.4. Statistical Analysis

Statistical analysis of the numerical data was performed to determine means and standard errors using Statistica software version 13. Significance of differences between means was determined by one-way analysis of variance (ANOVA) using Tukey’s t-test, for significance levels of 0.05 and 0.01.

## 3. Results

### 3.1. Chemical Composition of Oils

The chemical composition of oils is presented in Table 1. The flaxseed oil was much richer in PUFA (over 14%), particularly C_18:3_ ω-3 (over 86%), than the soybean oil, while the content of saturated fatty acids (SFA) in the flaxseed oil was over 40% lower than in the soybean oil. The ω-6/ω-3 ratio in the soybean oil was about 24 times as high as in the flaxseed oil. The total content of tocopherols and of all fractions (alfa, gamma, and delta) was much higher in the soybean oil than in the flaxseed oil (Table 1). Flaxseed oil exhibited a greater capacity to scavenge DPPH^•^ radicals than soybean oil (51.43 vs. 44.67) (Table 1).

### 3.2. The Blood Metabolites

The effect of soybean oil and flaxseed oil on plasma biochemical parameters is presented in Table 2. Horses receiving FO had significantly (*p* > 0.001) lower plasma levels of glucose, LDL and triacylglycerols, as well as lower ALT and ALP activity and a lower TC/HDL ratio (*p* = 0.01) than horses from the SO group. In turn, %HDL-TC and LDH activity were significantly higher in the FO group *(p* = 0.003, *p* > 0.001, respectively). There were no statistical differences in AST activity and content of TP, HDL, TC, P, Ca, and Mg.

### 3.3. Redox Status Parameters

The activities of antioxidant enzymes, i.e., SOD and CAT in the FO group were significantly (*p* > 0.001) higher than in the SO group (Table 3). In addition, FO contributed to an increase in levels of low-molecular-weight antioxidants, i.e., creatinine, vitamin C, zinc, and copper *(p* = 0.012, *p* > 0.001, *p* = 0.003 and *p* > 0.001, respectively). In contrast, plasma MDA level was lower in the FO group vs. the SO group (*p* = 0.004).

### 3.4. Haematological Parameters

The results of haematological analyses show that FO caused a significant increase in red-blood cell indicators, i.e., haemoglobin (*p* = 0.043), haematocrit (*p* = 0.011), MCV (*p* = 0.003) and MCH (*p* > 0.001) (Table 4). There was no differences between the treatments in total WBC and MID count, but the blood of horses receiving FO had a significantly higher lymphocyte count (*p* > 0.001) and lysozyme activity (*p* = 0.006), than the horses from the SO group. In turn, the NEU count in the blood of horses from the FO group was lower (*p* = 0.006) in comparison to the SO group.

## 4. Discussion

Analysis of blood parameters in horses is useful for evaluating their health status, nutrient requirements, fitness, and suitability for a given type of work [22]. Most of the haematological and biochemical parameters determined in the present study were within the wide range of reference values specified for horses [20,21], which demonstrates that the horses were in good health and condition.

The most commonly mentioned benefit of flaxseed oil is its effect on the blood lipid profile, and thus its major role in preventing cardiovascular disease [11]. In the case of horses, these diseases particularly affect older individuals, but are also encountered in younger animals [23]. In the present study, the inclusion of flaxseed oil in the diet of horses in place of soybean oil caused a substantial decrease in the level of TG and the atherogenic LDL fraction of cholesterol and an increase in the percentage of HDL-TC. An increase in the beneficial HDL fraction is one of the most important criteria in assessing a product as a hypocholesterolemic factor [24]. Flaxseed oil has also been shown to decrease the TC/HDL ratio, which is a good indicator of the risk of atherosclerosis and other cardiovascular complications [25]. The beneficial effect of flaxseed oil on lipid metabolism is linked to the presence of alpha-linolenic acid (ALA), which belongs to the family of ω-3 polyunsaturated fatty acids (PUFA), whose content in flaxseed oil may be about 57% [11]. In humans, ALA is metabolized, although to a very limited degree, to other representatives of this family of acids, i.e., to eicosapentaenoic acid (EPA) and docosahexaenoic acid (DHA) [26]. In horses, supplementation with ALA increases circulating EPA but not DHA [27]. Omega-3 PUFAs have the ability to reduce the plasma concentration of triacylglycerols by inhibiting their synthesis in the intestinal wall and liver [28]. Moreover, these acids reduce postprandial lipaemia by activating lipoprotein lipase, which is responsible for catabolism of very-low-density lipoproteins (VLDL) and chylomicrons [29]. Relatively recent experiments have led to the discovery of the molecular mechanism of the lipid-lowering effect of these acids. It was demonstrated that ALA may act as a signal molecule; via PPARα receptors it affects the expression of a number of genes associated with conversions of fatty acids, as well as other compounds [30]. Studies on rats with hypercholesterolemia have confirmed the beneficial effect of supplementation with flaxseed oil or flaxseeds on blood lipid indicators [11]. One of these compounds, secoisolariciresinol diglucoside (SDG), has the ability to lower LDL l and increase HDL [31]. Lignans are present mainly in flaxseeds, but flaxseed oil is also a source of these compounds, although their content is many times lower than that of whole seeds [32]. The presence of these compounds acting as estrogens may disrupt the metabolism of sex hormones in males and negatively affect reproductive indices. This was evidenced by the results of studies where flaxseeds were used, and not FO [33,34]. It should be mentioned that lignans are weak estrogens [35] and as mentioned earlier, their content in oil is much lower than in seeds, which minimizes the risk of adverse effects.

According to Lichtenstein and Schwab [36], the source of fat can affect glucose metabolism and its passage from the bloodstream to tissue. A low level of ω-3 PUFA and a high level of ω-6 PUFA in the phospholipids of the membranes of skeletal muscle cells favour the development of insulin resistance, which can result in type 2 diabetes [26]. Enrichment of the diet with ω-3 PUFA, e.g., by adding flaxseeds or flaxseed oil, may increase tissue sensitivity to insulin and prove effective in preventing diabetes. Promising results in this regard were obtained in an experiment on horses [37], in which insulin resistance is a significant problem. Insulin resistance in horses, which is linked to the development of diseases such as laminitis, osteochondrosis and metabolic syndrome, can result in serious production losses [38]. In the present study, horses receiving flaxseed oil in their diet had a significantly lower blood glucose level than those receiving soybean oil, which may have been due to its better utilization by tissues. It should be noted here that the ω-6 to ω-3 ratio in the flaxseed oil used in the experiment was 0.31, while the corresponding ratio in the soybean oil was many times that level, at 7.48. In addition to ALA, the hypoglycaemic effect may have been due in part to lignans in the oil, as according to Prasad [39], SDG also exhibits antidiabetic activity. It should be noted, however, that the horses were healthy, and the glucose level was within the range of reference values [20,21].

Analysis of the effect of flaxseed oil on liver profile markers in the blood of the horses revealed a decrease in the activity of ALT and to a lesser extent also AST. Aminotransferases ALT and AST are indicator enzymes of which increased levels in the blood suggest damage to the organs in which they are produced (increased ALT activity in particular indicates liver damage). A reduction in these enzymes in the blood, on the other hand, has no diagnostic significance [20,21]. It is difficult to interpret the significant increase in lactate dehydrogenase (LDH) activity in the blood of the horses receiving flaxseed oil. This is an indicator enzyme typical of muscle tissue, an increased level of which may indicate muscle damage. In this case, however, this can most likely be ruled out, given the values of other biochemical markers and the good health status of the animals.

Markers of kidney function in the blood include the levels of creatinine, urea, and uric acid. These compounds, alongside glutathione, bilirubin, and coenzyme Q, are included among endogenous low-molecular-weight antioxidants. The increase in creatinine in the blood of horses supplemented with flaxseed oil may thus have resulted from stimulation of antioxidant defence mechanisms. The increased level of ω-3 PUFA in flaxseed oil relative to soybean oil may have been the cause of increased oxidation reactions and free radical generation. An increase in antioxidant defence through increased synthesis of compounds inhibiting free radical reactions can be regarded as a kind of adaptive mechanism [40]. It is worth noting that the blood of horses receiving flaxseed oil had increased concentrations of antioxidants such as vitamin C and creatinine, but also of the enzymatic antioxidants catalase (CAT) and superoxide dismutase (SOD). The activity of these enzymes is highly specific and effective, and for this reason they are the main elements of the body’s antioxidant defence, while non-enzymatic mechanisms have only a supporting role [41]. Among antioxidant enzymes, SOD is considered an inducible enzyme, whose activity rises in response to an increase in the amount of the substrate, i.e., superoxide radical. Due to stimulation of various antioxidant defence responses, the concentration of the lipid peroxidation end product MDA in the blood of the horses receiving flaxseed oil was lower than in the horses receiving soybean oil. Another contributor may have been exogenous antioxidants present in flaxseed oil, such as lignans, plastochromanol-8, or peptides, phenolic acids, and carotenoids [8]. The most important antioxidants present in oilseeds include tocopherols, but their content in flaxseed oil is lower than in other vegetable oils with similar PUFA content [42]. Low content of tocopherols may be compensated for by the content of plastochromanol-8, which exhibits activity 1.5 times greater than that of α-tocopherol [43], the presence of lignans, and also peptides and proteins passing into the oil (despite filtration) from the seed pulp [44]. An experiment by Pilar et al. [1] compared the antioxidant activity of SDG, whole flaxseeds, and flaxseed oil in rats with metabolic syndrome. SDG proved most effective in reducing oxidative stress in the animals, but all of the supplements caused an increase in the activity of SOD, CAT and glutathione peroxidase (GPx). According to Naqshbandi et al. [45], who tested the ability of flaxseed oil to eliminate oxidative damage to the kidneys induced by anti-cancer therapy, it is largely the result of stimulation of SOD, CAT and GPx activity. In a previous study by Czech et al. [10], who analysed the effect of replacing soybean oil with flaxseed oil in the diet of turkeys, there was also an increase in SOD and CAT activity in the blood, as well as an increase in the FRAP value (due to an increase in the vitamin E concentration). The authors also noted an increase in the blood content of certain micronutrients (Cu, Fe and Zn) functioning as cofactors of antioxidant enzymes. The results of our study show that the increased SOD activity in the blood of horses receiving flaxseed oil was accompanied by an increase in the concentration of Zn and Cu ions. Apart from their significant role in enzymatic mechanisms, these metals also function as non-enzymatic antioxidants, and inadequate levels of them result in increased oxidative stress [46].

Another beneficial effect observed as a result of including flaxseed oil in the diet of horses was an improvement in the haematological parameters of the blood, i.e., an increase in Hb, HCT, MCV and MCH. Studies in healthy people [47] and patients with hyperglycaemia [48] have shown that the ω-3 PUFA in flaxseed oil or fish oil can effectively become incorporated in the phospholipids of the cell membranes of erythrocytes, which ensures their correct structure, and thus also their functions. Al-Zuhairy and Taher [49], who administered flaxseeds to chickens as a source of ALA, noted an increase not only in the RBC count, but also in the number of blood cells taking part in the immune response, i.e., leukocytes. No increase in the WBC count was observed in the horses receiving flaxseed oil, but changes were noted in the numbers of various types of leukocytes. There was a significant increase in the number of lymphocytes, accompanied by a decrease in that of neutrocytes. Apart from changes in the leukogram, the effect of flaxseed oil on the immune system of the horses may also be reflected in the increase in lysozyme activity, an important component of non-specific immunity. ALA and other ω-3 PUFAs are probably responsible for the immunomodulatory activity of flaxseed oil. By influencing the composition of membrane phospholipids of mononuclear cells, neutrophils, and platelets, they determine the number of eicosanoids generated [50]. These in turn are important mediators of inflammatory reactions; eicosanoids derived from ω-3 fatty acids function as anti-inflammatory agents, while those derived from ω-6 fatty acids act as pro-inflammatory factors [51].

## 5. Conclusions

The results indicate that flaxseed oil exerts a beneficial effect by stimulating antioxidant defence mechanisms and reducing the severity of oxidative stress. Flaxseed oil also improved the lipid profile and haematological parameters of the blood. Therefore, the introduction of flaxseed oil to the diet of horses in place of soybean oil appears justified, but this should be verified in further research including its effect on the gut microbiota as well as immunity indices and muscle metabolism.

## Figures and Tables

**Table 1:** Chemical composition of oils.

Item	Oil	
	Soybean	Flaxseed
Fatty Acids, %		
C_14:0_	0.11	0
C_16:0_	10.6	5.01
C_16:1_	0.09	0
C_17:0_	0.21	0.06
C_18:0_	3.76	3.73
C_18:1_ ω-9	23.7	21.51
C_18:1_ ω-7	1.61	0.68
C_18:2_ ω-6	51.54	16.2
C_18:3_ ω-3	6.89	52.2
C_22:0_	0.55	0.23
C_20:1_	0.28	0.12
C_20:0_	0.34	0.17
C_20:2_	0.03	0.00
C_20:3_	0.06	0.00
C_24:0_	0.23	0.09
ω-6/ω-3	7.48	0.31
SFA	15.8	9.29
MUFA	25.68	22.31
PUFA	58.52	68.4
Total tocopherol, mg 100 g^−1^	118.9	74.08
α-tocopherol	9.57	12
γ-tocopherol	78.42	47.98
δ-tocopherol	30.9	14.1
DPPH^•^, scavenging %	44.67	51.43

SFA—saturated fatty acids; MUFA—monounsaturated fatty acids; PUFA—polyunsaturated fatty acids; DPPH^•^ means stable free radical 2,2-diphenyl-1-picrylhydrazyl.

**Table 2:** Effect of dietary oils on biochemical parameters (mean ± SE) of the blood plasma of horses.

Item	Unit	SO	SE	FO	SE	*p*-Value	Reference Values [20,21]
GLU	mmol L^−1^	5.97	0.128	4.79	0.163	>0.001	3.5–6.0
TP	g L^−1^	66.36	2.61	61.73	4.54	0.370	55–75
AST	U L^−1^	351.0	10.08	341.0	10.01	0.116	138–409
ALT	U L^−1^	9.04	0.247	5.76	0.386	>0.001	3–25
ALP	U L^−1^	221.8	6.17	182.9	6.17	>0.001	109–315
LDH	U L^−1^	917.7	19.61	1322.1	19.61	>0.001	520–1480
Lipid Parameters							
TC	mmol L^−1^	2.62	0.093	2.37	0.180	0.882	1.3–2.8
HDL	mmol L^−1^	1.16	0.049	1.28	0.075	0.276	
LDL	mmol L^−1^	0.932	0.087	0.450	0.130	0.044	
%HDL-TC		45.43	1.85	58.32	1.85	0.003	
TC/HDL		2.24	0.089	1.73	0.089	0.010	
TG	mmol L^−1^	0.652	0.029	0.369	0.029	>0.001	0.1–0.7
Mineral Elements							
Phosphorus	mmol L^−1^	1.52	0.048	1.48	0.048	0.611	1.0–1.8
Manganese	mmol L^−1^	0.749	0.017	0.785	0.017	0.551	0.7–1.1
Calcium	mmol L^−1^	2.90	0.087	3.04	0.146	0.618	2.68–3.35

SO—control group with soybean oil; FO—experimental group with flaxseed oil; SE—standard errors; GLU—glucose; TP—total protein; AST—aspartate aminotransferase; ALT—alanine aminotransferase; ALP—alkaline phosphatase; LDH—lactate dehydrogenase; TC—total cholesterol; HDL—high-density lipoprotein cholesterol; %HDL-TC—percent of high-density lipoprotein cholesterol in total cholesterol; LDL low-density lipoprotein cholesterol; TG—triacylglycerols.

**Table 3:** Effect of dietary oils on redox status parameters (mean ± SE) of the blood plasma of horses.

Item	Unit	SO	SE	FO	SE	*p*-Value	Reference Values [20,21]
UA	µmol L^−1^	39.37	1.55	42.68	2.76	0.440	29.7–42.6
CREAT	µmol L^−1^	119.0	3.74	136.1	3.74	0.012	106.1–167.9
FRAP	mmol L^−1^	0.341	0.027	0.408	0.027	0.079	
Vitamin C	µmol L^−1^	0.210	0.006	0.268	0.006	>0.001	
MDA	µmol L^−1^	0.768	0.058	0.472	0.058	0.004	
SOD	U mL^−1^	1.89	0.100	2.98	0.103	>0.001	
CAT	U mL^−1^	2.51	0.105	3.49	0.111	>0.001	
Copper	µmol L^−1^	10.92	0.183	13.18	0.180	>0.001	
Zinc	µmol L^−1^	20.89	0.642	24.62	0.641	0.003	14.9–29.2
Iron	µmol L^−1^	15.29	0.428	15.53	0.400	0.731	13.1–25.1

SO, FO, SE—see Table 2; UA—uric acid; CREAT—creatinine; MDA—malondialdehyde; FRAP—total antioxidant potential; SOD—superoxide dismutase; CAT—catalase.

**Table 4:** Effect of dietary oils on haematological parameters (mean ± SE) of horses.

Item	Unit	SO	SE	FO	SE	*p*-Value	Reference Values [20,21]
Red blood cell parameters							
RBC	10^12^ L^−1^	7.88	0.271	8.36	0.267	0.250	6–10
Hb	mmol L^−1^	8.20	0.330	8.59	0.300	0.043	6.8–11.8
HCT	L L^−1^	0.342	0.094	0.399	0.089	0.011	0.32–0.53
MCV	fL	41.14	1.21	46.80	1.19	0.003	34–58
MCH	fmol	0.971	0.615	1.16	0.555	>0.001	8.1–11.8
MCHC	mmol L^−1^	20.76	0.407	20.50	0.400	0.177	19.2–23.0
White blood cell parameters							
WBC	10^9^ L^−1^	8.21	0.288	7.23	0.426	0.249	5.4–14.3
NEU	10^9^ L^−1^	5.09	0.276	3.23	0.406	0.006	4.0–7.8
LYM	10^9^ L^−1^	3.08	0.149	3.97	0.146	>0.001	1.5–5.0
MID	10^9^ L^−1^	0.036	0.005	0.051	0.003	0.081	
Lysozyme	mg L^−1^	0.574	0.040	0.726	0.053	0.006	

SO, FO, SE—see Table 2; RBC—red blood cell count; Hb—haemoglobin content; HCT—haematocrit; MCV—mean corpuscular volume; MCH—mean corpuscular haemoglobin; MCHC—mean corpuscular haemoglobin concentration; WBC—white blood cell count; NEU—neutrophils; LYM—lymphocytes; MID—the sum of monocytes, eosinophils and basophils.

## Abbreviation

%HDL-TC	percent of high-density lipoprotein cholesterol in total cholesterol
ALA	alpha-linolenic acid
ALP	alkaline phosphatase
ALT	alanine aminotransferase
AST	aspartate aminotransferase
BW	body weight
CAT	catalase
CREAT	creatinine
DHA	docosahexaenoic acid
DPPH^•^	means stable free radical 2,2-diphenyl-1-picrylhydrazyl
EPA	eicosapentaenoic acid
FID	flame ionization detector
FRAP	total antioxidant potential
GLU	glucose
GPx	glutathione peroxidase
Hb	haemoglobin content
HCT	haematocrit
HDL cholesterol	high-density lipoprotein cholesterol
HPLC	high-performance liquid chromatography
LDH	lactate dehydrogenase
LDL cholesterol	low-density lipoprotein cholesterol
LYM	lymphocytes
MCH	mean corpuscular haemoglobin
MCHC	mean corpuscular haemoglobin concentration
MCV	mean corpuscular volume
MDA	malondialdehyde
MID	the sum of monocytes, eosinophils, and basophils
MUFA	monounsaturated fatty acids
NEU	neutrophils
PN-EN ISO	Polish standard-European Standards International Organization for Standardization
PUFA	polyunsaturated fatty acids
RBC	red blood cell count
SDG	secoisolariciresinol diglucoside
SFA	saturated fatty acids
SOD	superoxide dismutase
TC	total cholesterol
TG	triacylglycerols
TP	total protein
UA	uric acid
WBC	white blood cell count
